# Attenuated Virulence and Genomic Reductive Evolution in the Entomopathogenic Bacterial Symbiont Species, *Xenorhabdus poinarii*

**DOI:** 10.1093/gbe/evu119

**Published:** 2014-06-05

**Authors:** Jean-Claude Ogier, Sylvie Pagès, Gaëlle Bisch, Hélène Chiapello, Claudine Médigue, Zoé Rouy, Corinne Teyssier, Stéphanie Vincent, Patrick Tailliez, Alain Givaudan, Sophie Gaudriault

**Affiliations:** ^1^INRA, UMR Diversité, Génomes et Interactions Microorganismes-Insectes (DGIMI), Montpellier, France; ^2^Université Montpellier 2, UMR Diversité, Génomes et Interactions Microorganismes-Insectes (DGIMI), France; ^3^INRA Toulouse Midi-Pyrénées, Unité MIA-T, Chemin de Borde Rouge, Castanet-Tolosan, France; ^4^CEA, Genoscope & CNRS, UMR 8030, Laboratoire d’Analyse Bioinformatique en Génomique et Métabolisme, Evry, France; ^5^Université Montpellier 1, UFR des Sciences Pharmaceutiques et Biologiques/UMR95 Qualisud, CIRAD-Persyst, France

**Keywords:** entomopathogenic bacteria, Lepidoptera, *Steinernema*, comparative genomics, regions of genomic plasticity, genomic deletion

## Abstract

Bacteria of the genus *Xenorhabdus* are symbionts of soil entomopathogenic nematodes of the genus *Steinernema*. This symbiotic association constitutes an insecticidal complex active against a wide range of insect pests. Unlike other *Xenorhabdus* species, *Xenorhabdus poinarii* is avirulent when injected into insects in the absence of its nematode host. We sequenced the genome of the *X. poinarii* strain G6 and the closely related but virulent *X. doucetiae* strain FRM16. G6 had a smaller genome (500–700 kb smaller) than virulent *Xenorhabdus* strains and lacked genes encoding potential virulence factors (hemolysins, type 5 secretion systems, enzymes involved in the synthesis of secondary metabolites, and toxin–antitoxin systems). The genomes of all the *X. poinarii* strains analyzed here had a similar small size. We did not observe the accumulation of pseudogenes, insertion sequences or decrease in coding density usually seen as a sign of genomic erosion driven by genetic drift in host-adapted bacteria. Instead, genome reduction of *X. poinarii* seems to have been mediated by the excision of genomic blocks from the flexible genome, as reported for the genomes of attenuated free pathogenic bacteria and some facultative mutualistic bacteria growing exclusively within hosts. This evolutionary pathway probably reflects the adaptation of *X. poinarii* to specific host.

## Introduction

Symbioses between microorganisms and animals are widespread in numerous ecological niches. Mutualistic symbiosis is based on mutual exploitation, in which each organism contributes to the interaction but receives a net positive benefit. The benefits are diverse and include a mutual influence on nutrition, defense, reproduction, and development (Chaston and Goodrich-Blair 2010). Multipartite microbial symbiosis involves long-term associations between three or more species, with at least two of the partners benefiting from the interaction (Hussa and Goodrich-Blair 2013). In the *Steinernema*–*Xenorhabdus* symbiotic interaction, soil entomopathogenic nematodes from the genus *Steinernema* are dependent on their intestinal bacterial symbiont, *Xenorhabdus* (*Enterobacteriaceae*), for colonization of the insects serving as their nutritional and reproductive niche. A nonfeeding soil-dwelling infective juvenile stage of the nematode penetrates the hemocel of the insect and releases the bacteria into the hemolymph. The bacterial symbiont helps to overcome insect immunity, kills the insect, and converts the cadaver into an essential source of food for nematode growth and development. Nematodes undergo several rounds of reproduction within the insect cadaver. When nematode density becomes too high and the nutrients derived from the cadaver are exhausted, the bacteria recolonize the nematodes, which then emerge from the insect cadaver into the soil, to search for a new host (Goodrich-Blair and Clarke 2007; Richards and Goodrich-Blair 2009; Nielsen-LeRoux et al. 2012). No free-living forms of *Xenorhabdus* have ever been isolated outside of the nematode host (Forst et al. 1997). Except the direct vectorization in the insect hemolymph, the benefit of the association to the bacterium has yet to be elucidated.

Since the 1980s, various species of entomopathogenic nematodes have been sold and used as effective biological control. agents for soil-inhabiting insects. Field and laboratory studies have demonstrated the importance of matching the appropriate nematode species with the particular pest targeted (Ehlers 2001). For example, the *Steinernema carpocapsae*–*Xenorhabdus nematophila* couple is virulent in various insect orders, and has been shown to be effective against *Pseudaletia unipuncta* (Lepidoptera), *Acheta domesticus* (Orthoptera), and *Plectrodera scalator* (Coleoptera). By contrast, the *St. glaseri*–*Xenorhabdus poinarii* couple is virulent principally in a few coleopteran species, such as *Popillia japonica* and *Cyclocephala hirta*, suggesting a potentially narrow host specificity (Wang et al. 1994; Converse and Grewal 1998; Rosa et al. 2002; Fallon et al. 2006).

Co-operation between the bacterial and the helminthic partner also differs between entomopathogenic couples. Under laboratory conditions, the bacterium or the nematode can be entomopathogenic alone. The injection of a dose of 100 cells of the bacteria *X. nematophila* and *X. bovienii* into larvae is lethal in diverse insects (Poinar and Thomas 1966; Forst et al. 1997; Ansari et al. 2003; Sugar et al. 2012). Aposymbiotic *St. carpocapsae* and *St. feltiae* nematodes lacking the *Xenorhabdus* symbiont can kill *Galleria mellonella* or *Tipula oleracea* although nematode reproduction is less efficient in the insect cadaver in the absence of the symbiont (Ehlers et al. 1997; Han and Ehlers 2000). These associations can, therefore, be considered as facultative for both partners under laboratory conditions. For example, a substantial body of molecular data has been accumulated on the factors enabling bacteria of species *X. nematophila* to adapt to the insect host in the absence of the nematode (Herbert et al. 2007; Nielsen-LeRoux et al. 2012). By contrast, co-operation is of much greater importance for the killing of insects in some bacterium-nematode complexes. In the *St. glaseri*–*X. poinarii* couple, the bacterial symbiont is avirulent or only weakly virulent when artificially injected into several insects (Akhurst 1986; Rosa et al. 2002; Ansari et al. 2003). No mortality is observed after the experimental infestation of *G. mellonella* with axenic *St. glaseri* nematodes (Akhurst 1986). However, a large proportion of *St. glaseri* nematodes are naturally aposymbiotic (Akhurst 1986). These contradictory features make it difficult to evaluate the facultative status of the *St. glaseri*–*X. poinarii* association.

Host-adapted bacteria have been described in both mutualistic and pathogenic symbioses. Obligatory mutualistic symbionts of insects (e.g., *Buchnera*, *Wigglesworthia*, etc.) live in specialized host organs (bacteriomes) and share a long-standing coevolutionary history with their host. They are vertically transmitted and display extreme genomic reduction. Facultative mutualistic symbionts are not strictly necessary for their host. They do not live exclusively in a specialized organ and undergo horizontal transfers between host strains or species (Dale and Moran 2006). These symbionts may be found in an active free-living stage (e.g., the squid symbiont *Vibrio fischeri*) or may grow exclusively within the hosts (e.g., the insect symbiont *Wolbachia*). *Wolbachia*, like some host-adapted pathogenic bacterial species, such as *Burkholderia mallei* and *Mycobacterium leprae,* displays a massive expansion of insertion sequences (IS), leading to pseudogene formation, chromosomal rearrangements mediated by recombination between IS and moderate genome downsizing. These features are considered to constitute the initial stages of a drastic reduction of genome size (Moran and Plague 2004; Gomez-Valero et al. 2007; Song et al. 2010). Finally, some host-adapted pathogenic bacteria, such as *My. tuberculosis* and the asymptomatic bacteriuria (ABU) strains of *Escherichia coli*, display moderate genome downsizing without massive IS expansion (Zdziarski et al. 2008; Veyrier et al. 2011). In such cases, the decrease in genome size probably results from the excision of mobile genetic elements.

We focused here on the pathogenic and genomic properties of the very poorly documented species *X. poinarii*. We compared the virulence of five *X. poinarii* strains by injecting them into the lepidopteran *Spodoptera littoralis*. We confirmed that all five strains tested had attenuated virulence. We sequenced the genomes of the *X. poinarii* G6 strain (*Xp_G6*) and the *X. doucetiae* FRM16 (*Xd*) strain, a closely related virulent strain. We showed that the genome of *Xp_G6* was much smaller (500–700 kb) than those of *Xd*, *X. nematophila* ATCC19061 (*Xn*) and *X. bovienii* SS-2004 (*Xb*), which have recently been sequenced and analyzed (Ogier et al. 2010; Chaston et al. 2011). This small genomic size is a key feature of the *X. poinarii* species resulting from reductive evolution, probably mediated by the deletion of regions of genomic plasticity (RGP). Thus, our study made it possible to compare the evolutionary history of the *X. poinarii* genome with that of other *Xenorhabdus* species.

## Materials and Methods

### Bacterial Strains, Media, Phenotypic Analyses, and Genomic DNA Extraction

All the bacterial strains used in this study are listed in table 1. *Xenorhabdus* strains were routinely grown in Luria–Bertani (LB) broth, 1.5% nutrient agar medium (GNO) or NBTA medium (GNO supplemented with 25 mg bromothymol blue and 40 mg triphenyl-2,3,4 tetrazolium chloride per liter) at 28 °C. Bacteria were stored at −80 °C in 16% glycerol (v/v). Genomic DNA was extracted as previously described (Gaudriault et al. 2006) and stored at 4 °C.

**Table 1:** List of *Xenorhabdus* Strains Used in This Study

Strain	Species	Nematode Host from Which Strain Was Isolated	Geographical Origin	Reference
G6 (ATCC 49121)	*X. poinarii*	*Steinernema glaseri*	USA (NC)	Akhurst 1986
AZ26	*X. poinarii*	*St. glaseri*	Portugal	Rosa et al. 2002
NC33	*X. poinarii*	*St. glaseri*	USA (NC)	Fischer-Le Saux, Arteaga-Hernandez, et al. 1999
SK72	*X. poinarii*	*St. glaseri*	USA (FL)	Fischer-Le Saux, Arteaga-Hernandez, et al. 1999
CU01	*X. poinarii*	*St. cubanum*	Cuba	Fischer-Le Saux, Arteaga-Hernandez, et al. 1999
ATCC19061	*X. nematophila*	*St. carpocapsae*	USA	Chaston et al. 2011
SS2004	*X. bovienii*	*St. jolietti*	USA (MO)	Chaston et al. 2011
FRM16	*X. doucetiae*	*St. diaprepesi*	Martinique	Fischer-Le Saux, Arteaga-Hernandez, et al. 1999

### Phylogenetic Analysis

Sequence alignment was generated and phylogenetic methods were performed as previously described (Tailliez et al. 2010, 2012). Briefly, for each bacterial strain, individual gene fragments (*recA*, 646 nucleotides; *gyrB*, 864 nucleotides; *dnaN*, 828 nucleotides; *gltX*, 913 nucleotides; and *infB*, 965 nucleotides) were aligned using MUSCLE (Edgar 2004) and then concatenated with the seaview platform (http://doua.prabi.fr/software/seaview, last accessed June 17, 2014). Ambiguously aligned blocks were removed by the Gblocks method (Castresana 2000) or the “Guidance” program (Penn et al. 2010). Maximum-likelihood analysis (phyML 3.0) was carried out with the general time-reversible model of substitution with gamma-distributed rate heterogeneity and a proportion of invariant sites determined for all five protein-coding sequences by jModelTest, to give the best fit to the data according the Akaike information criterion (Posada and Crandall 1998). MUSCLE, Gblocks, PhyML, and bootstrap values were obtained from the phylogeny.fr platform (Dereeper et al. 2008). Five *X. poinarii* strains, 23 type strains representative of *Xenorhabdus* species and the *Xb* strain, the genome of which has been sequenced, were included in this study. Three strains of *Photorhabdus* and one strain of *Proteus mirabilis* were used as closely related outgroups. The accession numbers of the individual genes used for building phylogenetic trees are listed in supplementary table S1, Supplementary Material online. The Enterobacteriaceae phylogenetic tree was constructed as described above, from the concatenated sequences of 12 conserved individual genes from the core genome (*infB, nusA, polA, pyrD, rpoB, valS, cysS, metK, purA, tpiA, smpB, secY*) of 47 *Enterobacteriaceae* strains. These genes belong to the defined set of 205 single-copy genes resistant to horizontal genetic transfer (HGT) and providing a reliable and consistent reconstruction of the phylogeny of γ-Proteobacteria (Lerat et al. 2003). These 12 genes were chosen for study on the basis of their homogeneous distribution along the length of the chromosome, and their similar model of DNA sequence evolution, as assessed with jModeltest (Posada and Crandall 1998). The nucleotide sequences used to construct the phylogenetic trees for Enterobacteriacae and *xaxA* were extracted from publicly available genomes.

### Insect Pathogenicity Assays

Bacteria were directly injected into two model insects: *Sp. littoralis* (Lepidoptera: Noctuidae) corn variant from Spain and *G. mellonella* (Lepidoptera: Pyralidae), as previously described (Sicard et al. 2004). For *Sp. littoralis*, all injections were performed on 1-day-old sixth-instar larvae that had been reared on an artificial diet (Poitout 1970) at 23 ± 1 °C, with a photoperiod of L16:D8 and a relative humidity of 40 ± 5%. For *G. mellonella*, all injections were performed on last-instar larvae reared at 28 °C in the dark with honey and pollen. *Xenorhabdus* strains were grown in LB broth (Difco) at 28 °C, with shaking, to exponential growth phase, corresponding to an optical density of 0.8 at 600 nm (Jenway Colorimeter). We injected 20 µl of bacterial suspension, containing 500–1,000 cells, into 20 larvae, with a Hamilton syringe. The surface of the insect larva was sterilized with 70% (v/v) ethanol before the injection of the bacteria into the hemocoel. The number of bacteria injected into the larvae was checked by plating serial dilutions on LB agar plates. Insect mortality was assessed at regular point times after injection, for the evaluation of LT_50_. At least four independent experiments were performed for each strain. For *Sp. littoralis* assays, statistical analysis was carried out with the Statistical Package for Social Science version 11.0.1 (SPSS, Chicago, IL), comparing individual survival times within each group.

### Sequencing and Assembly of the Whole Genomes

The complete genome sequences of *Xp_G6* and *Xd* were obtained by a mixture of Sanger capillary and new sequencing technologies. We first added about 23-fold coverage of 454 GSflx (Roche; www.roche.com, last accessed June 17, 2014) reads to Sanger reads, derived from a library with an insert fragment size of 10 kb. This library was constructed by the mechanical shearing of genomic DNA and insertion of the fragments generated into pCNS (pSU18-derived). Plasmid DNA was then purified and end-sequenced (5,745 reads for *Xp_G6* and 5,877 reads for *Xd*) by dye-terminator techniques, with ABI3730 sequencers (Applied Biosystems, Foster City, CA), resulting in approximately 1-fold coverage for both genomes. After assembly with Arachne (www.broadinstitute.org, last accessed June 17, 2014), to decrease the number of scaffolds, 5.6-fold coverage mate-paired 454 GSflx reads (with a library insert size of about 3 kb) were then added. The whole reads were assembled with Newbler (Roche) and validated via the Consed interface (www.phrap.org, last accessed June 17, 2014). For the finishing phase, we used primer walking on clones, polymerase chain reaction (PCR) and in vitro transposition technology (Template Generation System II Kit; Finnzyme, Espoo, Finland), generating 359, 162 and 533 additional reads, respectively, for *Xp_G6* and 701, 823 and 3,701 additional reads, respectively, for *Xd*. Illumina reads (36 bp) corresponding to a coverage of about 50-fold were mapped with SOAP (http://soap.genomics.org.cn, last accessed June 17, 2014) during the polishing phase, as previously described (Aury et al. 2008).

### PCR Amplification and the Sequencing of Nucleotidic Fragments

PCR amplifications targeting selected genomic regions were carried out, for analysis of the distributions of these regions in a panel of *Xenorhabdus* strains. Consensual pairs of primers (supplementary table S2, Supplementary Material online) were manually designed from clustalW alignments (http://multalin.toulouse.inra.fr/multalin/, last accessed June 17, 2014) of selected regions of *Xenorhabdus* reference genomes. Fragments with a predicted size of less than or greater than 3 kb were amplified with *Taq* polymerase (Invitrogen) or with the High Proof DNA Polymerase (BioRad), respectively, according to the manufacturer’s protocol. PCR amplifications were performed with a BioRad thermocycler (BioRad), and PCR products were analyzed by electrophoresis in an agarose gel. Amplicons were sequenced by MWG-Eurofins France.

### Pulsed-Field Gel Electrophoresis Analysis

Intact genomic DNA was extracted in agarose plugs as follows. Bacterial cells grown on nutrient agar plates were suspended in phosphate-buffered saline (GIBCO; Invitrogen) to a turbidity of 1.25 at 650 nm, included in 1.2% low-melting point agarose (SeaPlaque®GTG) solution (v/v) and lysed, as previously described (Jumas-Bilak et al. 1998). I-*Ceu*I (New England Biolabs) hydrolysis was performed as previously described (Teyssier et al. 2005). Pulsed-field gel electrophoresis (PFGE) was performed in a 0.8% agarose gel, in 0.5× Tris–borate–ethylenediaminetetraacetic acid buffer, at 4.5 V/cm and 10 °C, in a CHEF-DRII apparatus (BioRad). The separation of I-*Ceu*I fragments was optimized by using different electrophoresis conditions for fragments of different sizes: 1) a pulse ramp from 5 to 35 s for 24 h for fragments of less than 500 kb in size and 2) a pulse ramp from 150 to 400 s for 45 h for I-*Ceu*I fragments between 500 and 4,000 kb in size. The molecular markers used were the chromosomes of *Saccharomyces cerevisiae* and *Hansenula wingei* (BioRad).

### Genomic Analyses

#### Genome Annotation

Functional annotation was carried out with the tools of the MicroScope platform (Vallenet et al. 2013) and the annotated genome was implemented in the public XenorhabduScope database (https://www.genoscope.cns.fr/agc/microscope/home/index.php, last accessed June 17, 2014). We used specific tools for the annotation of specific gene families. The nonribosomal peptide synthetase (NRPS) and polyketide synthase (PKS) genes were predicted by the “2metDB” method (Bachmann and Ravel 2009) implemented at the MicroScope platform. Toxin–antitoxin (TA) systems were predicted with RASTA-Bacteria software (Sevin and Barloy-Hubler 2007) and we focused on predicted proteins with a domain characteristic of one of the nine most frequent TA system families: CcdA/CcdB, HicA/HicB, HigB/HigA, HipA/HipB, MazE/MazF, ParD/ParE, Phd/Doc, RelB/RelE, and VapB/VapC (Pandey and Gerdes 2005). We used the ISsaga platform (http://www-is.biotoul.fr/, last accessed June 17, 2014) to count IS (Varani et al. 2011).

#### Synteny Analysis

Whole-genome alignments were performed with the “Synteny Line Plot” tool available from the *MaGe* Platform (http://www.genoscope.cns.fr/agc/mage, last accessed June 17, 2014), which carries out a global comparison of two bacterial genomes on the basis of synteny results. The percentage of coding sequences (CDS) displaying synteny between the four genomes was calculated with the synteny statistic tool available from the *MaGe* Platform. The minimum size of the synteny groups was five genes.

#### Core and Flexible Genome Analysis

We used the SiLiX program of the MicroScope platform to cluster proteins into families of homologous sequences (MICFAM) (Miele et al. 2011). This program computes pan, core, and flexible genomes.

#### Analysis of Mobile Genetic Elements

RGP were sought in the four *Xenorhabdus* genomes (except for the plasmid of *Xn*). First, Prophinder was first used to detect prophages in the four *Xenorhabdus* genomes (Lima-Mendez et al. 2008) (http://aclame.ulb.ac.be/Tools/Prophinder/, last accessed June 17, 2014). We then used the RGPFinder web tool implemented via the MaGe annotation platform (http://www.genoscope.cns.fr/agc/mage, last accessed June 17, 2014) to identify GI (genomic islands) and RGP_sensu stricto_ (see Ogier et al. [2010] for detailed procedure). Briefly, *RGPFinder* searches for genomic regions (minimal size of 5 kb) displaying breaks in synteny between a query genome and a set of closely related genomes. If the regions displayed characteristics typical of foreign DNA acquired by HGT, such as compositional bias (GC% deviation, codon adaptation index) or tRNA, IS, integrase genes and genetic elements involved in DNA mobility, they were classified as GI. Regions without such features were classified as RGP_sensu stricto_. For the identification of integrative and conjugative elements (ICEs) in the *Xenorhabdus* genomes, we searched for genes encoding conjugation machinery, which consists of a relaxase, a T4SS and a type 4 coupling protein (Guglielmini et al. 2011). The ICE core was completed by searches for genes involved in 1) ICE replication, 2) DNA integration/excision, and 3) pilus biosynthesis (Seth-Smith et al. 2012).

#### Comparison of Gene Content

We used the MicroScope *Gene Phyloprofile* tool to identify sets of genes specific to *Xenorhabdus* genomes, with the following homology constraints: bidirectional best hit, minimal alignment coverage of 0.8, and amino acid sequence identity of 30%.

### Gene Remnant Identification

We analyzed gene remnants in the *Xp_G6* genome by first extracting protein sequences from the *Xd* genome present in the other two virulent strains, *Xn* and *Xb*, but absent from *Xp_G6* using results of the Gene Phyloprofile tool from the MicroScope platform and a custom-designed Perl Script. We then compared each of these proteins with the six-frame translations of the complete genome of *Xp_G6*, using the TBLASTN software, a sensitive method of searching for traces of partial coding regions not annotated in the *Xp_G6* genome. A gene was considered to be remnant in the *Xp_G6* genome if the corresponding TBLASTN results met the following criteria: HSP (high-scoring segment pair), including one to three different hits displaying at least 40% identity and with an *e* value <0.01.

## Results

### Phylogenetic Features of the Species *X. poinarii*

**Fig. 1.—:**
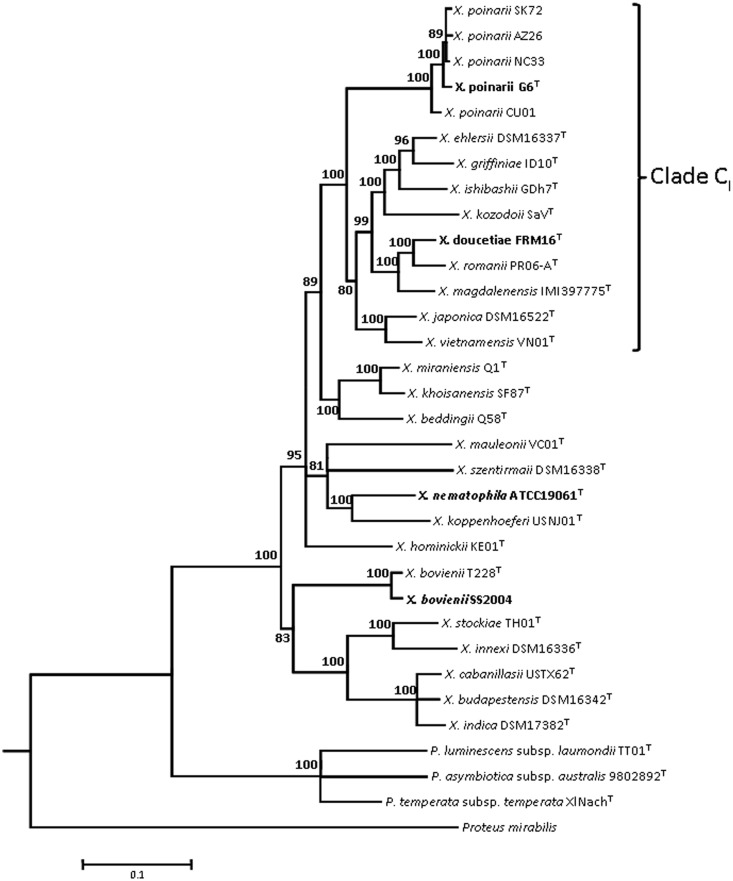
Maximum-likelihood phylogenetic tree showing the positions of *Xenorhabdus poinarii* strains within the genus *Xenorhabdus*. The analysis is based on five concatenated protein-coding sequences (*recA*, *gyrB*, *dnaN*, *gltX*, and *infB*). It was carried out with the GTR model of substitution, with a gamma-distributed rate heterogeneity and a proportion of invariant sites. *Photorhabdus* and *Proteus* sequences were used as outgroups. Bootstrap values (Felsenstein 1988) of more than 80% (from 100 replicates) are indicated at the nodes. Clade C_I_, which includes all the *X. poinarii* strains, is as previously described (Tailliez et al. 2010). The names of strains for which genomes have previously been sequenced or were sequenced in this study are indicated in bold italic and bold normal typescript, respectively. Bar: 10% divergence.

Strains AZ26, CU01, G6, NC33, and SK72 were previously classified within the species *X. poinarii* (Fischer-Le Saux, Viallard, et al. 1999; Tailliez et al. 2006) (table 1). We determined the phylogenetic position of the five strains within the genus *Xenorhabdus* with five concatenated protein-coding sequences: *recA*, *gyrB*, *dnaN*, *gltX*, and *infB* (fig. 1). The five strains clustered together on a clearly separate subbranch of clade C_I_ (Tailliez et al. 2010, 2012). Therefore, the species *X. poinarii* emerged from the clade C_I_ ancestor. The CU01 strain, which was located in a slightly different position, could be seen as lying at the edge of the species *X. poinarii,* as previously suggested by other authors (Fischer-Le Saux, Arteaga-Hernandez, et al. 1999; Tailliez et al. 2006).

### Pathology of the Species *X. poinarii*

The species *X. poinarii* has been described as weakly virulent following its direct injection into insect hemolymph (Akhurst 1986; Converse and Grewal 1998; Rosa et al. 2002; Ansari et al. 2003). We investigated the virulence of *X. poinarii* strains AZ26 (*Xp_AZ26*), CU01 (*Xp_CU01*), *Xp_G6*, NC33 (*Xp_NC33*) and SK72 (*Xp_SK72*) in insect larvae, by injecting 1,000 bacterial cells/larva directly into the hemocoel of *Sp. littoralis* and *G. mellonella*, these two insects being highly resistant and susceptible, respectively, to pathogenic bacteria. As a control, we used the strains *Xn* and *Xb*, two virulent *Xenorhabdus* reference strains, the genomes of which have been sequenced (Chaston et al. 2011). The two reference strains rapidly killed *Sp. littoralis* larvae, with an LT_50_ of 24–26 h, whereas the *X. poinarii* strains were strictly nonpathogenic (*P* < 0.05; table 2). By contrast, in *G. mellonella*, three *X. poinarii* strains (*Xp_G6*, *Xp_SK72,* and *Xp_CU01*) were found to be as virulent as the reference strains *Xn* and *Xb* (LT_50_ < 21 h). *Xp_NC33* was slightly attenuated (LT_50_ = 35 h) and *Xp_AZ26* was strictly nonpathogenic (table 2). We checked that weak virulence was not a feature common to the strains of phylogenetic clade C_I_, by also investigating the virulence of a type strain of this clade, *Xd*. This strain was as virulent as the reference strains, *Xn* and *Xb* in both insects (*P* < 0.05; table 2). In conclusion, the avirulence of the *X. poinarii* species in the highly resistant insect, *Sp. littoralis*, is a feature specific to this species.

**Table 2:** Pathogenicity of Eight Bacterial Strains Tested by Intrahemocoelic Injection into Last Instar-Larvae of *Spodoptera littoralis* (incubated at 23 °C) and *Galleria mellonella* (incubated at 28 °C)

Species	Strain	LT50 (h)^a^	
		*Sp. littoralis*^b^	*G. mellonella*
*Xenorhabdus poinarii*	G6	No mortality	<21
*X. poinarii*	AZ26	No mortality	No mortality
*X. poinarii*	NC33	No mortality	35
*X. poinarii*	SK72	No mortality	<21
*X. poinarii*	CU01	No mortality	<21
*X. nematophila*	ATCC19061	24	<21
*X. bovienii*	SS-2004	25–26	<21
*X. doucetiae*	FRM16	26–27	<21

^a^Mortality was recorded over 48 h; intrahemocoel injection of 1,000 bacterial cells/larva.

^b^Statistical analysis was carried out for each strain on at least four independent experiments (*P* < 0.05).

### Sequencing of the *Xp_G6* and *Xd* Genome Sequences

We investigated the genomic content of *X. poinarii* by sequencing the genome of *Xp_G6*, the type strain of the species isolated from the nematode *St. glaseri* G6 in North Carolina (Akhurst 1982, 1983; Akhurst and Boemare 1988). We also sequenced the genome of *Xd*, for a comparison of the genome of *Xp_G6* with a closely related pathogenic *Xenorhabdus* strain.

#### General Genome Features

The genomes of *Xp_G6* and *Xd* consist of circular chromosomes of 3,659,523 and 4,195,202 bp, encoding 3,715 and 3,974 proteins, respectively. In addition, *Xd* also harbors an 8,449-bp plasmid containing 12 protein-coding sequences displaying little similarity to the other CDS described, except for a putative sugar fermentation stimulation protein B (Ner-like protein) and a putative ParDE TA system (supplementary table S3, Supplementary Material online). The *Xp_G6* chromosome is clearly smaller (from 536 to 773 kb smaller) than the chromosome of the three virulent strains *Xn*, *Xb*, and *Xd* (table 3). The *Xp_G6* chromosome harbors fewer pseudogenes than the chromosomes of *Xn*, *Xb* and *Xd*, and this difference was particularly marked for the comparison with the 4.4 Mb chromosome of *Xn*, which is particularly rich in pseudogenes. It also has fewer repeated regions that usually serve as a substrate for chromosomal deletions and rearrangements (Treangen et al. 2009) than the chromosome of *Xn*, *Xb,* and *Xd*. The four strains have similar coding sequence densities (from 80% to 86%). *Xp_G6* and *Xd* contain 156 and 192 putative IS, respectively, a much smaller number than for *Xn* and *Xb* (436 and 369, respectively).

**Table 3:** Comparison of the Genomic Features in *Xenorhabdus nematophila* ATCC19061, *X. bovienii* SS-2004, *X. doucetiae* FRM16, and *X. poinarii* G6

Feature	*X. nematophila* ATCC19061		*X. bovienii* SS-2004	*X. doucetiae* FRM16		*X. poinarii* G6
	Chromosome	Plasmid	Chromosome	Chromosome	Plasmid	Chromosome
Size (bp)	4,432,590	155,327	4,225,498	4,195,202	8,449	3,659,522
G+C content (%)	44.19	45.97	44.97	45.71	45.09	44.55
CDS	4,299	175	4,260	3,974	12	3,715
Coding density (%)	80.52	79.62	85.64	85.93	50.21%	84.67
Mean CDS (bp)	860	711	850	933	464	856
Mean intergenic length (bp)	163	150	158	155	495	164
Repeated regions (%)	16.58%	17.99%	16.27%	16.18%	0	9.21%
Pseudogenes	99	4	58	45	0	38
IS	436	ND	369	192	ND	156
Phage genes	275 (6)	ND	437 (8)	157 (7)	0	377 (7)
rRNA operons	7	0	7	7	0	7
tRNAs	79	0	83	76	0	77
Accession number	FN667742	FN667743	FN667741	FO704550	FO704549	FO704551

Note.—ND, not determined.

#### Xenorhabdus Pan Genome, Core Genome, and Flexible Genome

**Fig. 2.—:**
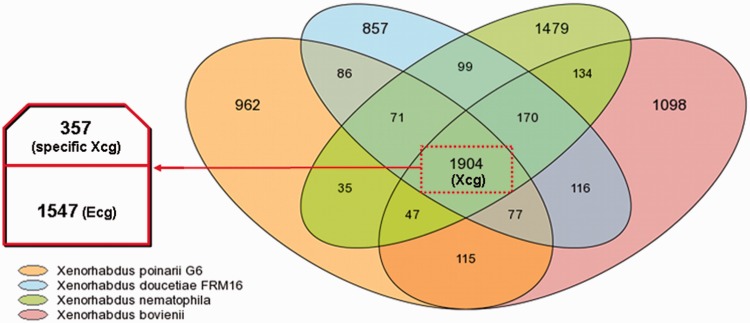
Venn diagram showing numbers of orthologous genes in the genomes of *Xenorhabdus nematophila* ATCC19061, *X. bovienii* SS-2004, *X. doucetiae* FRM16 and *X. poinarii* G6. The Xcg (1,904 gene families) is framed by red dashes, and includes the Ecg (1,547 gene families common to *Escherichia coli* K12) and the specific Xcg (357 gene families).

We analyzed the pan, core, and flexible genomes of the four *Xenorhabdus* genomes (fig. 2). The *Xenorhabdus* pan genome, corresponding to the total number of gene families present in *Xenorhabdus*, consists of 7,250 gene families. The *Xenorhabdus* core genome (Xcg), corresponding to the set of gene families common to the four *Xenorhabdus* strains, consisted of 1,904 gene families, or 40–50% of all the gene families present in each *Xenorhabdus* strains. We then introduced *Es. coli* strain K12, a commensal Enterobacteriaceae strain, into the analysis, which made it possible to identify an Enterobacteriaceae core genome (Ecg) of 1,547 gene families. The subtraction of the Ecg from the Xcg left us with 357 gene families that we considered to constitute the specific Xcg (see list in supplementary table S4, Supplementary Material online). The specific Xcg probably includes genes encoding factors essential for the *Xenorhabdus* lifestyle, particularly for symbiosis with the *Steinernema* and pathogenicity in insects. It encompasses many gene families previously described as encoding putative effectors of host interactions in the highly studied species *X. nematophila*: 1) factors potentially involved in hemocyte toxicity, such as the XhlA hemolysin, the RtxA toxin, the pore-forming fimbrial subunit MrxA, and enzymes involved in the synthesis of the lipopolysaccharide endotoxin; 2) enzymes required for the biosynthesis of rhabduscin, which inhibits phenoloxidase activity, an innate immune defense strategy of insects; 3) PrtS, PrtA, XlpA, EstA, PulA, extracellular enzymes probably involved in cadaver degradation; and 4) xenorhabdicin, a phage tail-like bacteriocin involved in intraspecies and interspecies competition within the nematode partner (Herbert et al. 2007; Chaston et al. 2011; Crawford et al. 2012). Interestingly, in addition to many genes encoding proteins of unknown functions, the specific Xcg also contains genes potentially involved in iron metabolism and transport, sodium transport, histidine and thiamine metabolism, and resistance to tellurium.

The flexible genome (corresponding to the subtraction of the Xcg from the pan genome) consists of gene families absent from at least one of the genomes compared. The flexible genome of each strain accounts for 42%, 44%, 53% and 49% of the total numbers of gene families in the *Xp_G6*, *Xd*, *Xn* and *Xb* genomes, respectively. The flexible genome is rich in strain-specific gene families (23–36%) mostly annotated as conserved genes of unknown function, orphan genes or genes associated with mobile and extrachromosomal elements, suggestive of probable acquisition by horizontal gene transfer.

#### Regions of Genomic Plasticity

**Fig. 3.—:**
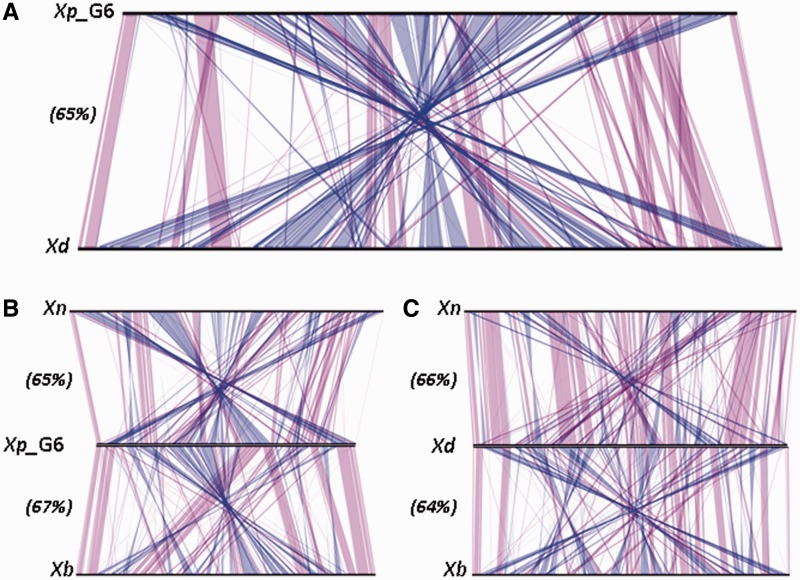
Whole-genome sequence alignments between *Xenorhabdus* genomes. The line plots were obtained with the results for synteny between (*A*) *X. poinarii* G6 (*Xp_G6*) and *X. doucetiae* FRM16 (*Xd*); (*B*) *Xp_G6*, *Xn*, and *Xb*; (*C*) *Xd*, *Xn*, and *Xb*. Matches between synteny groups occurring on the same strand are shown in purple; matches between synteny groups occuring on the opposite strand are shown in blue. Numbers in brackets indicate the percent of CDS in synteny for each whole-genome alignment.

For the vizualization of strain-specific regions, we generated a whole-genome alignment of the sequences of *Xp_G6* and *Xd* (fig. 3*A*). Despite belonging to closely related species, *Xp_G6* and *Xd* displayed numerous shuffled regions, with synteny conservation for only 65% of the CDS. The large-scale genome rearrangements revealed by synteny comparison were not correlated with differences in genome sizes: whole-genome alignments of *Xp_G6* and *Xd* with *Xb* and *Xn* (fig. 3*B* and *C*) displayed similar rearrangement patterns and similar percentages of CDS in synteny (64–67%). Genome rearrangements are, therefore, widespread within the genus *Xenorhabdus*, as previously described for *Xb* and *Xn* (Ogier et al. 2010).

The large-scale genome rearrangements revealed by genome synteny comparison may result from recombination events (horizontal gene transfer, duplications, inversions, deletions) in the flexible genome. The flexible genome is often structured into RGP, which contain mobile genetic elements, such as genomic islands (GI) and prophage loci, and hypervariable segments, hereafter referred as RGP_sensu stricto_ (Ogier et al. 2010). We identified the RGP of the *Xb* and *Xn* genomes by comparing these genomes with a set of Enterobacteriaceae genomes, using the RGPFinder tool (Ogier et al. 2010). In this study, we carried out a new analysis with the four *Xenorhabdus* genomes as the set of genomes for comparison, leading to the identification of 57, 67, 73 and 79 RGP in the *Xp_G6*, *Xd*, *Xb* and *Xn* genomes, respectively. *Xp_G6* had the smallest number of RGP, accounting for 34% of the entire genome, versus 40–43% for the other three *Xenorhabdus* genomes considered (table 4 and supplementary table S5, Supplementary Material online). No integral RGP was conserved in all four genomes and, as previously described, only subregions of RGP, named modules, were conserved. This suggests that modules are the true units of plasticity in *Xenorhabdus* genomes (Ogier et al. 2010).

**Table 4:** Number and Classification of RGP in the *Xenorhabdus nematophila* ATCC19061, *X. bovienii* SS-2004, *X. doucetiae* FRM16, and *X. poinarii* G6 Genomes as a Function of Their Genetic Composition (numbers in brackets indicate the percentage of total genome size)

	Number of Prophages (%)	GI (%)	RGP_sensu stricto_^a^ (%)
*X. poinarii* G6	7 (7.3)	25 (16.4)	25 (8.8)
*X. doucetiae* FRM16	7 (3.7)	27 (19)	33 (19.3)
*X. bovienii* SS-2004	8 (7.1)	24 (16)	41 (14.3)
*X. nematophila* ATCC19061	6 (6.2)	30 (21.5)	43 (15.6)

^a^RGP that are not prophages or GI.

We searched for ICEs among the RGP. By contrast to what has been shown for the closely related strain *Photorhabdus luminescens* TT01, but similarly with the ICEHIn1056 of *Haemophilus influenza* strain 1056, the potential *Xenorhabdus* ICEs consisted of only a remnant of the pilus synthesis locus, an entire or partial *pilL* gene (supplementary table S6, Supplementary Material online). This part is not essential for ICE self-mobilization (Seth-Smith et al. 2012). Thus, each of the four strains has one entire chromosomal ICE without a pilus synthesis locus and one partial chromosomal ICE, lacking the other features of canonical ICEs. Moreover, *Xn* harbors an entire ICE with no pilus synthesis locus on its megaplasmid and a partial copy of it on the chromosome. This last feature probably results from integration of the *Xn* plasmid into the chromosome, followed by gene loss and plasmid immobilization. In addition to ICEs, we also classified RGP into GI, prophages, or RGP_sensu stricto_. We found similar numbers of GI in *Xp_G6*, *Xd*, *Xb*, and *Xn* (25, 27, 24, and 30, respectively; table 4). GI were generally located within conserved integration hot spots throughout the genome (supplementary table S5, Supplementary Material online), but gene content was rarely conserved within the GI. The ProPhinder tool allowed us to classify some RGP as prophages (Lima-Mendez et al. 2008; Ogier et al. 2010) (table 4). The four genomes were found to harbor similar numbers of prophage regions. Two P2-related phage clusters have already been described in *Xb* (*xbp1* and *xbp2*) and *Xn* (*xnp1* and *xnp2*), and *xbp1* and *xnp1* have been shown to encode the components of a phage tail-like bacteriocin (Morales-Soto et al. 2012). Likewise, we described *xdp1* and *xdp2* in *Xd* and *xpp1* in *Xp_G6* (supplementary table S5, Supplementary Material online). Finally, RGP_sensu stricto_, which did not display features of foreign DNA acquired by HGT, accounted for only 9% of the entire *Xp_G6* genome, whereas they accounted for 14–19% of the other three genomes (table 4).

### Genomic Content of *Xp_G6* and *Xd* and Comparative Analysis with *Xb* and *Xn* Genomes

We screened the content of the *Xp_G6* and *Xd* genomes and compared it with that of the *Xb* and *Xn* genomes by two approaches. We first searched for genes or loci potentially involved interactions with the host and/or environment on the basis of their annotation. We then systematically searched for genes specifically absent from *Xp_G6* and present in the other genomes (supplementary table S7, Supplementary Material online). The genomic regions or genes with a remarkable distribution in the four genomes are listed in table 5.

**Table 5:** Selection of Genomic Regions or Genes of Interest Regarding the Bacterial Life-Cycle in the *Xenorhabdus poinarii* G6 (*Xp_G6*), *X. doucetiae* FRM16 (*Xd*), *X. nematophila* ATCC19061 (*Xn*), and *X. bovienii* SS2004 (*Xb*) Genomes

Function	Product or Function	Gene or Locus			
		*Xn*	*Xb*	*Xd*	*Xp_G6*
T5SS (Tps)	XhlA/B	XNC1_4556-4555	XBJ1_0258-0258	XDD1_3768-3767	XPG1_0219-0220
	Putative CDI	Absent	XBJ1_1975-79	XDD1_1117-1119	Absent
	Others	XNC1_3685-3689	Absent	Absent	Absent
		XNC1_3564-3565			

Hemolysin	XaxAB	XNC1_3766-3767	XBJ1_1710-1711	XDD1_0809-0810	Absent

Insecticidal toxin	PirAB	XNC1_1143-1142	Absent	XDD1_2939-2940	XPG1_1629-1630
	Txp40	XNC1_1129	Absent	Absent	Absent
	Mcf2	XNC1_2028	Absent	XDD1_1049 (truncated)	Absent
	Mcf-like	XNC1_2265	XBJ1_2410	Absent	Absent
	Tc	3 complete loci, 2 partial loci^a^	1 complete locus, 4 partial loci^a^	Absent	Absent

NRPS–PKS	PAX-peptide synthesis	XNC1_2781-2784	XBJ1_2151-2155	XDD1_2664-2669	Absent
	Unknown metabolite synthesis	XNC1_2037-2040	XBJ1_1966-1968	XDD1_2281-2289	Absent

Amino acid-related compounds catabolism	Choline catabolism and transport	XNC1_1244-1247	XBJ1_3308-3305	XDD1_1182-1185	Absent
	Arginine and amino-butyrate metabolism	XNC1_2270-2274	XBJ1_0079-0085	XDD1_1920-1927	Absent
	Hydroxyphenylacetic acid catabolism	XNC1_0446-0449	XBJ1_3600	XDD1_0415	Absent
		XNC1_0810-0823	XBJ1_0867-0875	XDD1_1012-1026	Absent
			XBJ1_3599-3560 (partial)		
	Phenylalanine and phenylacetic acid catabolism	XNC1_3619-3621	XBJ1_3555-3557	XDD1_0655-0657	Absent
		XNC1_4614-4627	XBJ1_0117-0128	XDD1_3917-3928	Absent
	Tyrosine catabolism	XNC1_2243-2245	XBJ1_2348-2352	XDD1_2132-2135	Absent

Regulators	Two-component system YehU/YehT	XNC1_0512-0513	XBJ1_0375-0376	XDD1_0487-0488	Absent
	Quorum sensing regulator LuxS	XNC1_1265	XBJ1_3281	XDD1_1203	Absent
	Putative RcsA activator	XNC1_1652-1653	XBJ1_1898	XDD1_2386	Absent
	Transcriptional repressor for phenylacetic acid degradation	XNC1_4631	XBJ1_0116	XDD1_0107	Absent

Entire TA systems (type II^b^)	CcdAB	XNC1_0081-0082	Absent	Absent	Absent
	HipBA	XNC1_4231-4232	Absent	XDD1_0152-0153	Absent
	MazEF	XNC1_0471-0472	XBJ1_0440-0442	XDD1_0556-0557,	Absent
				XDD1_3836-3837	
	Doc–Phd	XNC1_0202-0203	XBJ1_4379-4380	XDD1_0111-0112	Absent
	RelBE	XNC1_1940-1941	XBJ1_3187-3188	Absent	Absent
	VapBC	XNC1_0417-0418,	Absent	XDD1_0524-0525,	Absent
		XNC1_4632-4633		XDD1_0577-0578	

Note.—Tps, two partner system; CDI, contact-dependent inhibitor.

^a^For further description, see Chaston et al. (2011).

^b^Predicted after a RASTA analysis (Sevin and Barloy-Hubler 2007) and from selection of nine families described in *Escherichia coli* or *Yersinia pestis* (CcdA/CcdB, HicA/HicB, HigA/HigB, HipA/HipB, MazE/MazF, ParD/ParE, Phd/Doc, RelE/RelB, VapB/VapC).

#### Secretion Systems

We explored the secretion potential of the four *Xenorhabdus* strains. The four genomes have nearly similar numbers of T1SS (21, 23, 20 and 18 for *Xn*, *Xb*, *Xd* and *Xp_G6*, respectively). As previously reported (Chaston et al. 2011), they possess genes encoding the Sec pathway, but they do not encode a T2SS to mediate the crossing of the outer membrane. Unlike the genomes of genus *Photorhabdus*, *Xenorhabdus* genomes have no genes encoding a T3SS, confirming the divergence between *Xenorhabdus* and *Photorhabdus* in terms of lifestyle (Chaston et al. 2011). We identified two T4SS loci in the *Xb*, *Xd* and *Xp_G6* genomes and four T4SS loci in the *Xn* genome, components of entire or partial ICEs (see above). Two T6SS loci were present in the *Xn*, *Xb* and *Xp_G6* genomes, and *Xd* was found to carry one additional copy. Finally, the distribution of loci for the T5SSs was particularly marked. The T5SS consists of a transported protein, TpsA, and a channel-forming protein, TpsB, the sole accessory protein devoted to the secretion of TpsA. All four genomes were found to contain a locus encoding XhlA–XhlB, which has been shown to be involved in the export of the XhlA hemolysin responsible for insect virulence in *Xn* (Cowles and Goodrich-Blair 2005). Only the *Xp_G6* genome lacked all the other T5SS systems (table 5).

#### Insecticidal Toxins, Cytotoxins, and Hemolysins

*Xenorhabdus* produces an array of insecticidal toxins (Hinchliffe et al. 2010). PirAB (Duchaud et al. 2003; Waterfield et al. 2005) is encoded by the *Xn*, *Xd* and *Xp_G6* genomes, whereas the Tpx40 toxin (Brown et al. 2006) is encoded by only the *Xn* genome. None of the *Xenorhabdus* strains encoded the Mcf1 toxin potentially responsible for triggering apoptosis in insect cells via a BH3-like N-terminal domain (Daborn et al. 2002; Dowling et al. 2007), but the *Xp_G6* was the only strain with no Mcf ortholog, Mcf2 or Mcf-like sequence (Waterfield et al. 2003). Interestingly, neither *Xd* nor *Xp_G6* was found to possess loci encoding proteins of the toxin complex (Tc) family (Waterfield et al. 2001). These high-molecular weight proteins have two effects: 1) an oral effect, due to the targeting and disruption of the intestinal epithelium of the lepidopteran *Manduca sexta* (Bowen et al. 1998) and 2) a phagocytosis-inhibiting effect on insect cells due to the modification of actin and Rho GTPases through ADP-ribosyltransferase activity (Lang et al. 2010; Lango and Clarke 2010). The *tc* genes are located in GI that were probably acquired by HGT (Waterfield et al. 2002; Ogier et al. 2010). The absence of *tc* loci from the genomes of *Xd* and *Xp_G6*, both of which belong to phylogenetic clade C_I_ (fig. 1), strongly suggests either gene loss or an absence of HGT for *tc* loci in the bacterial ancestor of phylogenetic clade C_I_. Interestingly, each *Xenorhabdus* strain possesses a different cocktail of insecticidal toxins, correlated with phylogenetic status rather than with virulence status. Moreover, the paucity of insecticidal genes in phylogenetic cluster I, which contains both virulent and avirulent strains, argues against a major role for insecticidal toxins in the virulence process.

*Xenorhabdus* also counteracts cellular immunity by producing cytotoxins and hemolysins (Nielsen-LeRoux et al. 2012). The *xaxAB* locus of the *X. nematophila* F1 strain, which encodes a binary pore-forming cytotoxin with apoptotic and necrotic activities in mammalian and insect cells (Vigneux et al. 2007), was absent only from the *Xp_G6* genome (table 5). The XaxA and XaxB proteins are probably required for tissue degradation in the cadaver and for efficient subsequent nematode reproduction (Jubelin et al. 2011).

#### NRPS and PKS

*Xenorhabdus* protects the insect cadaver from other organisms that might seek to use it as food, by synthesizing an array of secondary metabolites, including antibacterial molecules synthesized by large, multimodular enzymes: the NRPS and PKS (Bode 2009). By searching for NRPS and PKS domains, we identified 16, 13, 12 and 10 loci potentially encoding NRPS/PKS enzymes in the genomes of *Xn*, *Xb*, *Xd* and *Xp_G6*, respectively. Nevertheless, these similarities in the number of loci conceal considerable differences between the four genomes. Indeed, the number of NRPS/PKS modules, the functional units of the multimodular NRPS–PKS enzymes, was found to be significantly smaller in the *Xp_G6* genome (21 modules, 106 kb) than in the *Xb*, *Xd* and *Xn* genomes (56–79 modules, 253–413 kb). Furthermore, the *pax* locus, encoding NRPS enzymes involved in the synthesis of PAX peptides, which are lysine-rich antifungal cyclolipopeptides (Gualtieri et al. 2009; Fuchs et al. 2011), and an undescribed NRPS locus, weakly similar to a *Pseudomonas syringae* locus, were specifically absent from the present *Xp_G6* genome, but present in the other three *Xenorhabdus* genomes (table 5). This genomic pattern highlights the low potential of *Xp_G6* for the synthesis of secondary metabolites. These metabolites have a wide range of bioactive properties and have been reported to be involved in antimicrobial activities, cytotoxic activity, and immunomodulation in entomopathogenic bacteria (Gualtieri et al. 2009; Park et al. 2009; Vallet-Gely et al. 2010; Fuchs et al. 2011; Stein et al. 2012; Theodore et al. 2012). Their absence from *Xp_G6* may limit the capacity of this bacterium to kill the insect on its own.

#### Catabolism of Amino Acid-Related Compounds

A striking feature of the *Xp_G6* genome is the lack of genes encoding proteins involved in the catabolism of amino acid-related compounds: 1) choline and glycine betaine, 2) arginine and amino-butyrate, and 3) aromatic amino acid-related metabolites (supplementary table S7, Supplementary Material online, and table 5). Both primary and secondary metabolisms are required for optimal colonization of the nematode by *Ph. luminescens* and *Xn*, but not for bacterial virulence in insects (Martens et al. 2005; Orchard and Goodrich-Blair 2005; Lango and Clarke 2010; Easom and Clarke 2012). Further studies should provide new insight into the possible involvement of such metabolic clusters in *X. poinarii* pathogenicity.

#### TA Systems

TA (toxin-antitoxin) systems consist of two closely linked genes, encoding a stable toxin and a labile antitoxin. TA systems are involved in stabilizing genomic regions: when the TA locus is lost, the unstable antitoxin protein disappears first, causing cell death (Van Melderen and De Bast 2009). Additional roles in stress response and/or cell quality control were also recently described (Schuster and Bertram 2013). We identified 42, 12, 37 and 7 genes encoding products with antitoxin or toxin domains in *Xn*, *Xb*, *Xd* and *Xp_G6*, respectively, but intact TA loci (pairs of colocalized toxin and antitoxin genes) were totally absent from *Xp_G6* genome (table 5). Surprisingly, this feature seems to be a general feature of obligate intracellular organisms, whereas free-living slowly growing prokaryotes have a large number of such loci (Pandey and Gerdes 2005).

### Small Genome Size and Genomic Reduction, a General Feature of the Species *X. poinarii*

#### Small Genome Size

**Fig. 4.—:**
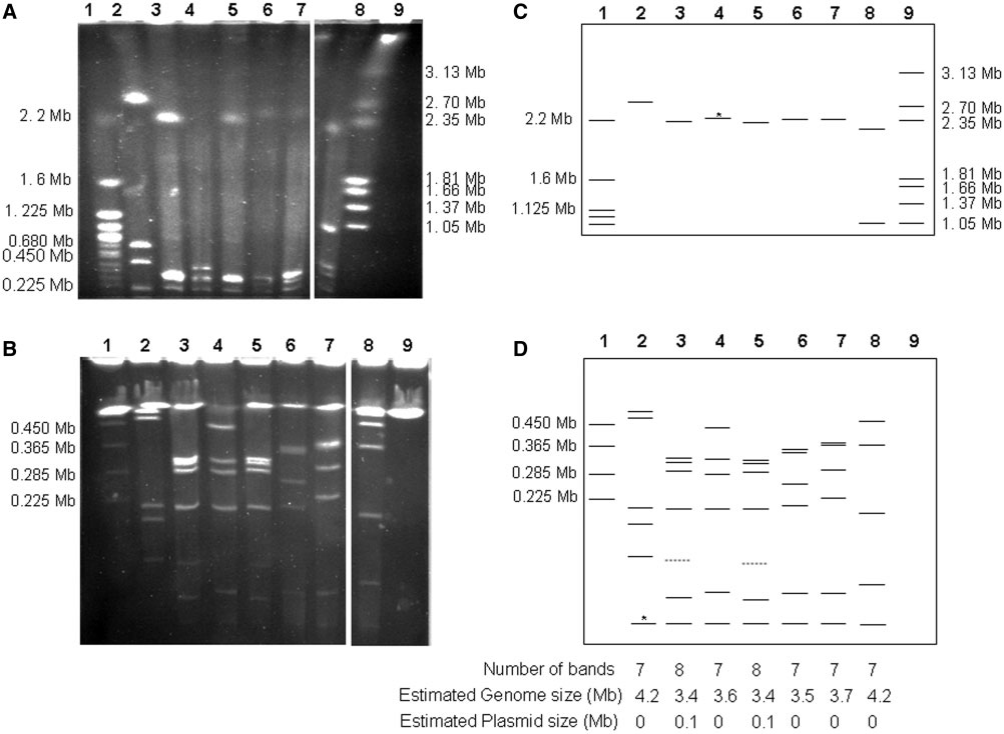
Estimation of *Xenorhabdus poinarii* strains genome size by PFGE of I-*Ceu*I-hydrolyzed genomic DNA. The separation of I-*Ceu*I fragments was optimized by using different electrophoresis conditions for fragments of different sizes: (*A*) a pulse ramp from 150 to 400 s for 45 h for I-*Ceu*I fragments between 500 and 4,000 kb in size; (*B*) a pulse ramp from 5 to 35 s for 24 h for fragments of less than 500 kb in size. Schematic representations of the I-*Ceu*I PFGE patterns under two sets of migration conditions, making it possible to separate fragments from 500 to 4,000 kb in size (*C*) and fragments from 10 to 500 kb in size (*D*), were also shown. Lane 1: *Saccharomyces cerevisiae* (strain 972h); lane 2: *X. bovienii* SS-2004; lane 3: *X. poinarii* AZ26; lane 4: *X. poinarii* G6; lane 5: *X. poinarii* SK72; lane 6: *X. poinarii* CU01; lane 7: *X. poinarii* NC33; lane 8: *X. doucetiae* FRM16; lane 9: *Hansenula wingei* (strain YB-4662-VIA). Dashed bands around 120 kb in strains *Xp_AZ26* (lane 3) and *Xp_SK72* (lane 4) correspond to fragments with a lower staining intensity, probably plasmids. *Although these bands are difficult to see on the gel photography, there were directly distinguishable on the gel and their sizes were confirmed by the theorical I-*Ceu*I pattern of the genome sequences of *X. bovienii* SS-2004 and *X. poinarii* G6. Fragment and genome sizes of the four unsequenced *X. poinarii* strains were evaluated with the *X. poinarii G6*, *X. bovienii* SS-2004, and *X. doucetiae* FRM16 genomes used as a reference (lanes 2, 4, and 8) and molecular weight ladders (lanes 1 and 9).

We investigated whether small genome size was a feature particular to the *Xp_ G6* strain or a general feature of the species *X. poinarii*, by examining the whole genome architecture of the other four *X. poinarii* strains (*Xp_SK72*, *Xp_AZ26*, *Xp_NC33*, and *Xp_CU01*) by I-*Ceu*I genomic macrorestriction. I-*Ceu*I specifically cleaves the eubacterial 23S rRNA gene of the *rrn* operon (Liu and Sanderson 1995). Based on the four *Xenorhabdus* and all the Enterobacteriaceae genome sequences, we expected to obtain seven I-*Ceu*I fragments. The number and sizes of the I-*Ceu*I fragments in the *X. poinarii* strains were analyzed by PFGE, with migration conditions allowing the separation of fragments from 10 to 4,000 kb (fig. 4). In total, seven DNA bands (ranging from 40 to 2,200 kb in size) were resolved in the gel runs for *X. poinarii* strains, except for strains *Xp_AZ26* and *Xp_SK72,* for which eight bands were observed. However, the bands of about 120 kb in size obtained for *Xp_AZ26* and *Xp_SK72* were probably not I-*Ceu*I hydrolysis fragments, corresponding instead to plasmid DNA, given that they were stained less intensely than the other bands (Teyssier et al. 2005). Finally, PFGE analysis of the *Xp_SK72*, *Xp_AZ26*, *Xp_NC33*, and *Xp_CU01* strains showed that these strains had genomes ranging in size from 3,400 to 3,700 kb. A small genome is, therefore, a general feature of the species *X. poinarii*.

#### Decay of Isolated Genes

We searched for gene remnants in the *Xp_G6* genome, by TBLASTN comparisons of the *Xd* proteins against the *Xp_G6* genome. We found only 24 remnants of *Xd* genes in *Xp_G6* (indicated in supplementary table S7, Supplementary Material online). In *Xd*, these genes are not clustered together in the same area of the genome. They are instead, spread throughout the genome.

#### Excisions within RGP: Example of the xaxAB Locus

**Fig. 5.—:**
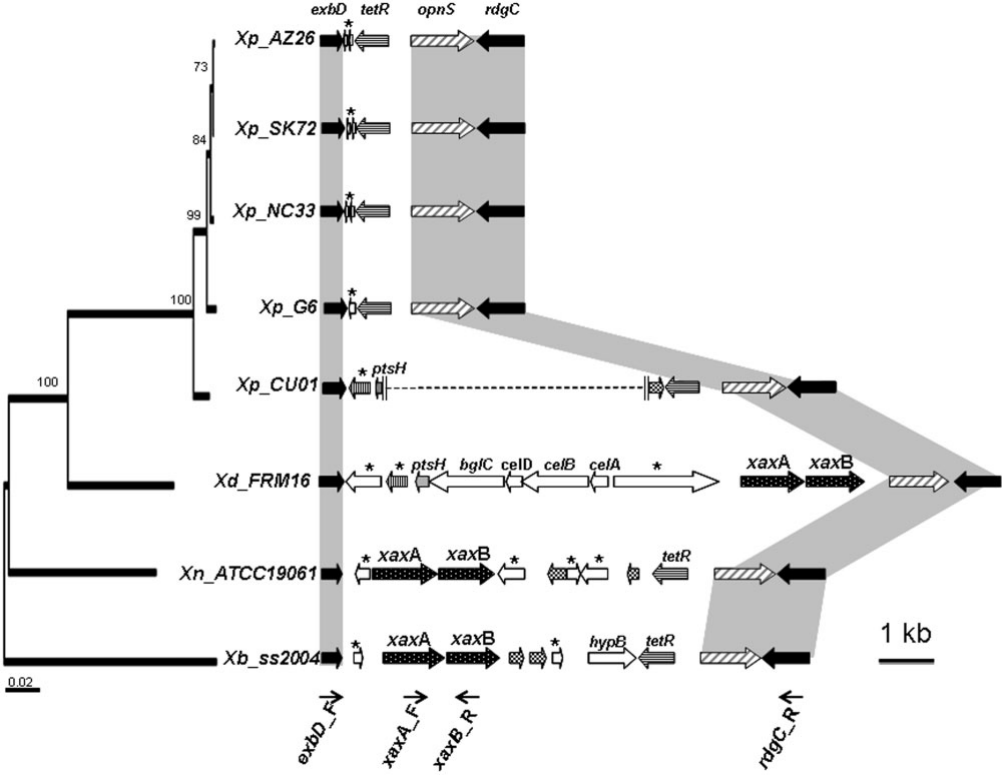
The *xaxAB* locus, its genomic context and its shuffling point *exbD*/*rdgC* in the *X. doucetiae* FRM16 (*Xd*), *X. nematophila* ATCC19061 (*Xn*), *X. bovienii* SS-2004 (*Xb*), *X. poinarii* G6 (*Xp_G6*), AZ26 (*Xp_AZ26*), NC33 (*Xp_NC33*), SK72 (*Xp_SK72*), and CU01 (*Xp_CU01*) genomes. The large arrows represent individual ORFs, and the names of the genes are indicated above the arrows. Genes encoding proteins of unknown function are marked with an asterisk. Orthologous genes are indicated by arrows in the same color. Black and chequered arrows represent core-genome genes and transposase genes, respectively. The thin arrows indicate the binding sites of the primers used for PCR amplification. The vertical parallel lines indicate the end of the sequenced area and the dotted lines represent an unsequenced genomic region. The cladogram was obtained by the maximum-likelihood phylogenetic analysis of five concatenated protein-coding sequences (*recA*, *gyrB*, *dnaN*, *gltX*, and *infB*), as already described in figure 1. The accession numbers of the sequences of the subsequent amplicons are HG934736 (strain AZ26), HG934737 (strain NC33), HG934738 (strain SK72), HG934739 and HG934740 (strain CU01).

The *xaxAB* locus encodes a hemolysin (see above) and is specifically absent from the *Xp_G6* strain. In *Xd*, *Xn,* and *Xb*, the *xaxAB* locus is embedded within RGP_sensu stricto_ (RGP14, RGP64, and RGP28, respectively), a class of RGP specifically underrepresented in the *Xp_G6* genome. RGP14, RGP64, and RGP28 are located at the same shuffling point, flanked by the genes of the core genome *exbD* and *rdgC*. In the *Xp_G6* genome, the genomic content between the *exbD* and *rdgC* genes has been significantly reduced, with the presence of only *tetR*, *opnS* and one small gene encoding a protein of unknown function (fig. 5). We investigated the presence of *xaxAB* genes in other *X. poinarii* strains, by using pairs of primers to amplify the genomic content within the *exbD*/*rdgC* shuffling point (*exbD*_F/*rdgC*_R). As a control, we first checked that the observed sizes of the amplicons matched the theoretical sizes, for the four sequenced genomes. For *Xp_AZ26*, *Xp_NC33,* and *Xp_SK72*, the size of the amplicon obtained from the sequences between *exbD* and *rdgC* was similar to that for *Xp_G6* (about 4 kb) and sequencing of the PCR fragments revealed a similar genomic organization in all four strains (fig. 5). Surprisingly, a 10-kb fragment was obtained from the *Xp_CU01* genome. Sequencing of the extremities of the regions of the *Xp_CU01* amplicon showed the conservation of some *Xd* genes, with a shuffled genomic organization and the presence of a transposase gene, highlighting progressive genomic erosion in *Xp_CU01*. The *xaxAB* locus was not found in the position observed in *Xd* nor in that observed in *Xb/Xn,* in *Xp_CU01*. We checked that the *xaxAB* locus was not present elsewhere in the *X. poinarii* genomes, by PCR amplification with the *xaxA*_F/*xaxB*_R primer pair on the five *X. poinarii* strains (data not shown).

**Fig. 6.—:**
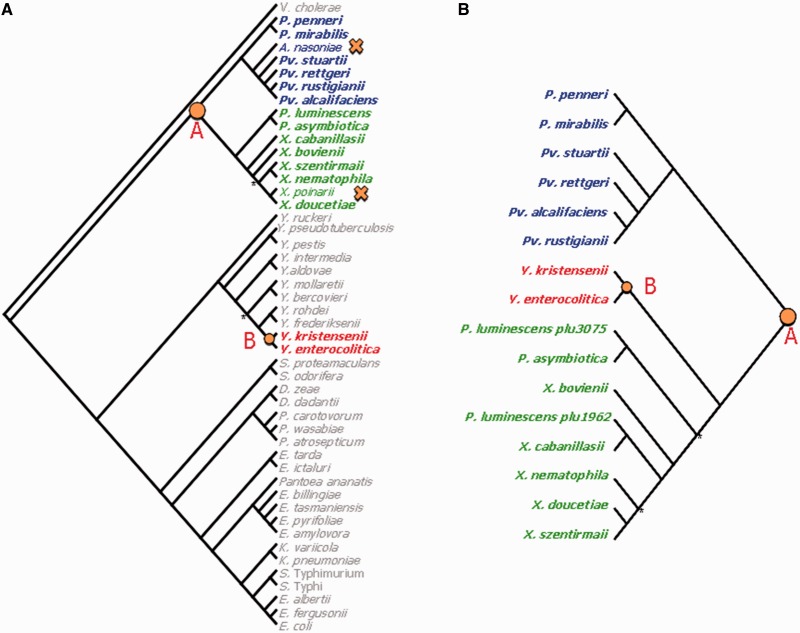
Analysis of the evolutionary history of the *xaxAB* locus by a comparison of topology between an Enterobacteriaceae tree and a *xaxA* gene tree. (*A*) Enterobacteriaceae phylogenetic tree based on a maximum-likelihood (ML) analysis of 12 core concatenated protein-coding sequences (*infB, nusA, polA, pyrD, rpoB, valS, cysS, metK, purA, tpiA, smpB, secY*). *Vibrio cholerae* sequences were used as the outgroup. Nodes are supported by bootstrap values of more than 93%, unless marked with an asterisk. (*B*) Phylogenetic tree based on ML analysis of the *xaxA* gene. Nodes are supported by bootstrap values of more than 86%, unless marked with an asterisk. Node A, the bacterial ancestor of the *Providencia–Proteus–Photorhabdus–Xenorhabdus* clade, which probably contained the *xaxA* gene. Node B, bacterial ancestor of the *Yersinia kristensenii* and *Y. enterocolitica* species, to which the *xaxA* gene was probably transferred horizontally. Crosses, probable deletions of the xaxA gene. *Vibrio cholerae* 16961: NC_002501; *Prot. penneri* ATCC35198: PRJNA54897; *Prot. mirabilis* HI4320: NC_010554; *Arsenophonus nasoniae* DSM15247: PRJNA185551; *Prov. stuartii* ATCC25827: PRJNA54899; *Prov. rettgeri* DSM1131: PRJNA55119; *Prov. rustigianii* DSM 4541: PRJNA55071; *Prov. alcalifaciens* DSM30120: PRJNA55119; *Ph. luminescens* TT01: NC_005126.1; *Ph. asymbiotica* ATCC43949: NC_012962; *X. cabanillasii* JM26: CBXE010000001-CBXE010000496; *X. bovienii* SS-2004: NC_013892; *X. szentirmaii* DSM16638: CBXF010000001-CBXF010000164; *X. nematophila* ATCC19061: NC_014228.1; *X. poinarii* G6: FO704551; *X. doucetiae* FRM16: FO704550; *Y. ruckeri* ATCC297473: PRJNA55249; *Y. pseudotuberculosis* IP31758: NC_009708; *Y. pestis* CO92: NC_003143; *Y. intermedia* ATCC29909: PRJNA54349; *Y. aldovae* ATCC35236: PRNJA35243; *Y. mollaretii* ATCC43969: PRJNA54345; *Y. bercovieri* ATCC43970: PRJNA54343; *Y. rohdei* ATCC43380: PRJNA55247; *Y. frederiksenii* ATCC33641: PRJNA54347; *Y. kristensenii* ATCC33638: PRJNA55245; *Y. enterocolitica* 8081: NC_008800; *Serratia proteamaculans* 568: NC_0098332; *Se. odorifera* DSM4582: PRJNA40087; *Dickeya zeae* 1591: NC_012912; *Dickeya dadantii* 586: NC_013592; *Pectobacterium carotovorum* PC1: NC_012917; *Pe. wasabiae* WPP163: NC_013421; *Pe. atrosepticum* SCRI1043: NC_004547; *Edwarsiella tarda* EIB202: NC_013508; *Edwarsiella ictulari* 93-146: NC_012779.2; *Pantoea ananatis* LMG20103: NC_013956; *Erwinia billingiae* At-9b: NC_014837; *Er. tasmaniensis* Eb661: NC_014306; *Er. pyrifoliae* Ep1/96: NC_02214; *Er. amylovora* ATCC49946: NC_013971; *Klebsiella variicola* At-22: NC_013850; *K. pneumoniae* 342: NC_011283; *Salmonella enterica* Typhimurium LT2: NC_003197; *Sal. enterica* Typhi CT18: AL513382; *Escherichia albertii* TW07627: PRJNA55089; *Es. fergusonii* ATCC35469T: NC_011740; *Es. coli* K12: NC_000913).

We tested the hypothesis that a deletion event occurred during *X. poinarii* speciation, by reconstructing the evolutionary history of the *xaxAB* locus within the Enterobacteriaceae family. We built and compared the topologies of an Enterobacteriaceae phylogenetic tree based on 12 housekeeping genes (fig. 6*A*) and a *xaxA* phylogenetic tree (fig. 6*B*) The Enterobacteriaceae tree grouped the genera into two clades: *Providencia*–*Proteus–Photorhabdus*–*Xenorhabdus* on the one hand and *Yersinia–Serratia–Dickeya–Edwarsiella–Erwinia–Klebsiella*–*Escherichia* on the other. We found that *xaxA* orthologs were present within 1) all the members of the *Providencia–Proteus–Photorhabdus–Xenorhabdus* clade other than the species *Arsenophonus nasoniae* and *X. poinarii*, and 2) only two *Yersinia* species in the other clade. These results suggest that the *xaxA* gene was present in the genome of the bacterial ancestor of the *Providencia–Proteus–Photorhabdus–Xenorhabdus* clade (node A in fig. 6*A*), from which it was transferred horizontally to the bacterial ancestor of *Yersinia kristensenii* and *Yersinia enterocolitica* species (node B in fig. 6*A*). The most parsimonious hypothesis explaining the absence of *xaxA* from *A. nasoniae* and *X. poinarii* would be the deletion of the locus (crosses in fig. 6*A*). *Arsenophonus nasoniae* infects the parasitic wasp *Nasonia vitripennis* and is responsible for the son-killer trait in wasps (Wilkes et al. 2011). Interestingly, like *X. poinarii*, *A. nasoniae* has a significantly smaller (3.6 Mb) genome than its closest relatives, the genera *Proteus* and *Providentia* (4–5 Mb), and this genome is not particularly rich in phage genes or transposons (Darby et al. 2010).

## Discussion

*Xenorhabdus* bacteria are fascinating models for studies of the mechanisms and evolution of symbioses, because they are both mutualistic symbionts in nematodes and pathogenic symbionts in insects. In recent years, *X. nematophila* has been widely analyzed, and many molecular and genomic data are now available for this species (Herbert et al. 2007; Nielsen-LeRoux et al. 2012). Several studies have focused on another species, *X. bovienii* (Chaston et al. 2011, 2013; Kim et al. 2012; Morales-Soto et al. 2012; Sugar et al. 2012). We report here the first analysis of genomic data for the species *X. poinarii*, which belongs to a phylogenetic group (clade C_I_), different from that of *X. nematophila* and *X. bovienii*. We showed that all the studied strains of *X. poinarii* had attenuated virulence following their experimental injection into insects. Furthermore, our genomic analysis revealed that a small genome was a general feature of the species *X. poinarii*. This feature is not typical of the phylogenetic group, because the closely related pathogenic *Xenorhabdus* strain, *Xd*, from clade C_I_ (Tailliez et al. 2010), has a genome with a size similar to those of *X. nematophila* and *X. bovienii* ones.

The small size of the genomes in the species *X. poinarii* may reflect either an ancestral state or a recent divergent evolution toward a small genome. In the first hypothesis, all *Xenorhabdus* strains would have originated from an ancestor with a small genome. In this scenario, *X. poinarii* would be the only species to have retained a small genome size, with all the others species experiencing genome expansion. However, the evolutionary scenario inferred from the phylogenetic topology based on five genes of the Xcg is not consistent with this hypothesis, because *X. poinarii* does not occupy a basal position in this phylogeny (fig. 1). According to the second hypothesis, the ancestor of the genus *Xenorhabdus* had a large genome and deletions have occurred specifically in *X. poinarii*. We observed both a slight gene decay and a paucity of RGP_sensu stricto_ in *X. poinarii* (supplementary table S7, Supplementary Material online, and table 4). We thus assume that the RGP_sensu stricto_ (i.e., hypervariable regions of the flexible genome [Ogier et al. 2010]) may have undergone deletion events in the genome of *X. poinarii*. We therefore propose that the genomes of *X. poinarii* strains have undergone a reduction with respect to those of other *Xenorhabdus* genomes, through the excision of genomic blocks from the flexible genome. As an illustration of how such deletion events could occur, we reconstructed the evolutionary history of the *xaxAB* locus (fig. 6)*,* which is embedded within RGP_sensu stricto_ in the larger genomes of *Xn*, *Xb*, and *Xd* (Ogier et al. 2010). Genomic excision is the most parsimonious hypothesis explaining the absence of the *xaxAB* locus from the *Xp-G6* genome. Several examples of similar deletions have already been reported in bacteria with smaller genomes and weaker virulence than other strains from the same taxon. In *Es. coli*, the ABU strains have smaller genomes than virulent strains. These strains display frequent point mutations and IS element-mediated deletions in the *fim* genomic cluster, which is responsible for fimbrial synthesis and the virulence of uropathogenic *Es. coli* strains (Zdziarski et al. 2008). Likewise, a 77-kb genomic region encoding methionine biosynthesis enzymes, T3SS effectors, and T4SS is deleted in the hypoaggressive *Ralstonia solanacearum* strain IPO1609. This region contains no features of GI or prophages. Its absence leads to a loss of pathogenicity (Gonzalez et al. 2011).

The compact structure and paucity of nonfunctional sequences in most prokaryotic genomes can generally be accounted for by an inherent deletional bias (Mira et al. 2001; Kuo and Ochman 2009). Host-adapted symbionts (intracellular or niche-restricted) generally have smaller genomes than the free-living bacteria from which they were derived (Moran 2002; Klasson and Andersson 2004). This evolution toward reduced genomes in host-adapted symbionts may be due to genetic drift (Mira et al. 2001; Silva et al. 2001; Nilsson et al. 2005). Indeed, because of sequestration in the host or the occurrence of major lifecycle stages within host, genome of host-adapted bacterial symbionts has reduced opportunity for counterbalancing deletional bias by HGT compared with free-living bacteria. Moreover, the restriction to specific hosts also promotes small bacterial population size, asexuality and population bottleneck for transmission, favoring the persistence of slightly deleterious mutations (Muller’s ratchet). The accumulation of these mutations entails a fitness cost to the bacterium and leads to DNA loss (McCutcheon and Moran 2012). The hallmarks of early stages of genetic drift-driven genomic reduction are an inordinately large number of pseudogenes, a low coding capacity, a high levels of transposable elements, phage-derived sequences, and a massive expansion of IS elements (Toh et al. 2006; Gavotte et al. 2007; Song et al. 2010; Leclercq et al. 2011). Positive selection may also be a significant driver of reductive genome evolution (Koskiniemi et al. 2012). In free-living bacteria growing on a restricted resources in a constant environment, positive selection minimizes the material costs of cellular replication, by reducing genome length (streamlining) (Giovannoni et al. 2005). Large-scale deletions of accessory genes may also be beneficial in a selective environment (Lee and Marx 2012). Comparisons of the large genomes of *Xd*, *Xn,* and *Xb* with the small genome of *Xp_G6* highlighted the deletion processes, by revealing large deletions spanning multiple genes (RGP_sensu stricto_) and small deletions of few nucleotides (gene remnants), together with equivalent coding capacity and the presence of similar numbers of GI, prophages, IS elements, pseudogenes, and phage genes (tables 3 and 4) in the four genomes. The features of the small *Xp_G6* genome are therefore rather consistent with a mechanism of selection-driven gene loss in the flexible genome than with a mechanism of genomic reduction dominated by a genetic drift. However, we cannot totally exclude the possibility that genome reduction was also promoted by a population bottleneck. Indeed, as a large proportion of *St. glaseri* nematodes is naturally aposymbiotic (Akhurst 1986), the transmission of *X. poinarii* to the next generation of *St. glaseri* nematodes would involve only a small bacterial population.

The *Xenorhabdus* lifecycle is characterized by a combination of pathogenic and mutualistic lifestyles and the routine, alternate infection of two kinds of invertebrate hosts. The reduced genome of *X. poinarii* does not prevent the bacterial/nematode symbiosis from having a lifestyle similar to that of other *Xenorhabdus* species. What is the evolutionary and ecological significance of genomic reduction in *X. poinarii*? Selection-driven genome reduction in mutualistic and pathogenic bacteria often results from a greater reliance on the host (Moran et al. 2008). We demonstrated the avirulence of *X. poinarii*, through direct bacterial injections into two lepidopteran insect species (table 2), as previously reported for several species from the Lepidoptera and Coleoptera (Akhurst 1986; Converse and Grewal 1998; Rosa et al. 2002; Ansari et al. 2003). It is possible that this phylogenetic bacterial group displays greater insect specificity than other *Xenorhabdus* species. However, no insects susceptible to direct injections of *X. poinarii* have yet been identified. Alternatively, the bacterial functions necessary for virulence following direct bacterial injection present in other *Xenorhabdus* species but absent from *X. poinarii* might be complemented by the nematode partner. Further studies are required to determine the possible role of such complementation in insect virulence.

In conclusion, this first genomic study on the species *X. poinarii* provides insight into the mechanisms underlying genomic erosion in symbiotic bacteria. In addition, our comparison of the genomes of this avirulent species with those of other *Xenorhabdus* species paves the way for the identification of new candidate virulence factors in the genus *Xenorhabdus*.

## Supplementary Material

Supplementary tables S1–S7 are available at *Genome Biology and Evolution* online (http://www.gbe.oxfordjournals.org/).

Supplementary Data
