# Supplementary material for: Attenuated Virulence and Genomic Reductive Evolution in the Entomopathogenic Bacterial Symbiont Species, Xenorhabdus poinarii
Source: Genome Biol Evol. 2014 Jun 5;6(6):1495–513. doi: 10.1093/gbe/evu119 (PMC4079199; doi:10.1093/gbe/evu119)
Supplement: Supplementary Data [file supp_6_6_1495__index.html]

Attenuated virulence and genomic reductive evolution in the entomopathogenic bacterial symbiont species, Xenorhabdus poinarii — Attenuated Virulence and Genomic Reductive Evolution in the Entomopathogenic Bacterial Symbiont Species, Xenorhabdus poinarii — Supplementary Data 

# Attenuated Virulence and Genomic Reductive Evolution in the Entomopathogenic Bacterial Symbiont Species, *Xenorhabdus poinarii*

## Supplementary Data

files

**Files in this Data Supplement:**

- Supplementary Data - xls file
- Supplementary Data - xls file
- Supplementary Data - xls file
- Supplementary Data - xls file
- Supplementary Data - xls file
- Supplementary Data - docx file
- Supplementary Data - xls file
