# Supplementary material for: Attenuated Virulence and Genomic Reductive Evolution in the Entomopathogenic Bacterial Symbiont Species, Xenorhabdus poinarii
Source: Genome Biol Evol. 2014 Jun 5;6(6):1495–513. doi: 10.1093/gbe/evu119 (PMC4079199; doi:10.1093/gbe/evu119)
Supplement: Supplementary Data [file supp_evu119_Table_S6.docx]

**Table S6**: List of integrative conjugative elements (ICEs) in the four sequenced genomes of *Xenorhabdus*.

| **ICE type** | **Xn** | **Xb** | **Xd** | **Xp_G6** |
| --- | --- | --- | --- | --- |
| Entire chromosomal ICE | **XNC1_3444- XNC1_3598**   - Replication region: present - Pilus region: absent - T4SS: present - Integration region: present | **XBJ1_3367- XBJ1_3411**   - Replication region: present - Pilus region: absent - T4SS: present - Integration region: absent | **XDD1_1065- XDD1_1132**   - Replication region: present - Pilus region: absent - T4SS: present - Integration region: present | **XPG1_2881- XPG1_2939**   - Replication region: present - Pilus region: absent - T4SS: present - Integration region: present |
| Partial chromosomal ICE | **XNC1_3639- XNC1_3681**   - Replication region: present - Pilus region: absent - T4SS: present - Integration region: absent | **XBJ1_1459- XBJ1_1564**   - Replication region: present - Pilus region: absent - T4SS: present - Integration region: present | **XDD1_1375- XDD1_1399**   - Replication region: present - Pilus region: absent - T4SS: partial - Integration region: absent | **XPG1_2573- XPG1_2646**   - Replication region: partial - Pilus region: absent - T4SS: present - Integration region: partial |
| Entire ICE on the megaplasmid | **XNC1_p0001- XNC1_p0071**   - Replication region: present - Pilus region: absent - T4SS: present - Integration region: present |  |  |  |
| Partial chromosomal ICE (partial copy of the ICE located on the megaplasmid) | **XNC1_2347- XNC1_2387**   - Replication region: partial - Pilus region: absent - T4SS: partial - Integration region: absent |  |  |  |

Xn: *Xenorhabdus nematophila* ATCC19061; Xb: *Xenorhabdus bovienii* SS-2004; Xd : *Xenorhabdus doucetiae* FRM16; Xp_G6: *Xenorhabdus poinarii* G6
